# A novel scoring system based on common laboratory tests predicts the efficacy of TNF-inhibitor and IL-6 targeted therapy in patients with rheumatoid arthritis: a retrospective, multicenter observational study

**DOI:** 10.1186/s13075-017-1387-9

**Published:** 2017-08-11

**Authors:** Jin Nakagawa, Yoshinobu Koyama, Atsushi Kawakami, Yukitaka Ueki, Hiroshi Tsukamoto, Takahiko Horiuchi, Shuji Nagano, Ayumi Uchino, Toshiyuki Ota, Mitsuteru Akahoshi, Koichi Akashi

**Affiliations:** 1 Department of Medicine and Biosystemic Science, Kyushu University Graduate School of Medical Sciences, Fukuoka, 812-8582 Japan; 2 Japanese Red-Cross Okayama Hospital, Center for Autoimmune Diseases, Division of Rheumatology, 2-1-1 Aoe, Kita-ku, Okayama 700-8607 Japan; 3 Department of Immunology and Rheumatology, Nagasaki University Graduate School of Biomedical Sciences, Nagasaki, 852-8501 Japan; 4 Rheumatic and Collagen Disease Center, Sasebo Chuo Hospital, Sasebo, 857-1195 Japan; 5 Department of Rheumatology, Shin-Kokura Hospital, Kitakyushu, 803-8505 Japan; 6 Department of Internal Medicine and Clinical Immunology, Kyushu University Beppu Hospital, Beppu, 874-0838 Japan; 7 Center for Rheumatic Diseases, Iizuka Hospital, Iizuka, 820-8505 Japan

**Keywords:** Rheumatoid arthritis, BDMARDs (biologic agents), Cytokines, IL-6, TNF

## Abstract

**Background:**

Currently, although several categories of biological disease-modifying antirheumatic drugs (bDMARDs) are available, there are few data informing selection of initial treatment for individual patients with rheumatoid arthritis (RA). Therefore, tumor necrosis factor inhibitor (TNF-i) and tocilizumab (TCZ) are treated as equivalent treatments in the recent disease management recommendations. We focused on two anticytokine therapies, TCZ and TNF-i, and aimed to develop a scoring system that predicts a better treatment for each RA patient before starting an IL-6 or a TNF-i.

**Methods:**

The expression of *IL-6* and *TNF-α* mRNA in peripheral blood from 45 newly diagnosed RA patients was measured by DNA microarrays to evaluate cytokine activation. Next, laboratory indices immediately before commencing treatment and disease activity score improvement ratio after 6 months in 98 patients treated with TCZ or TNF-i were retrospectively analyzed. Some indices correlated with TCZ efficacy were selected and their cutoff values were defined by receiver operating characteristic (ROC) analysis to develop a scoring system to discriminate between individuals more likely to respond to TCZ or TNF-i. The validity of the scoring system was verified in these 98 patients and an additional 228 patients.

**Results:**

There was significant inverse correlation between the expression of *IL-6* and *TNF-α* mRNA in newly diagnosed RA patients. The analysis of 98 patients revealed significant correlation between TCZ efficacy and platelet counts, hemoglobin, aspartate aminotransferase, and alanine aminotransferase; in contrast, there was no similar correlation in the TNF-i group. The cutoff values were defined by ROC analysis to develop a scoring system (1 point/item, maximum of 4 points). A good TCZ response was predicted if the score was ≥2; in contrast, TNF-i seemed to be preferable if the score was ≤1. Similar results were obtained in a validation study of an additional 228 patients. If the case scored ≥3, the good responder rates of TCZ/TNF-i were 75.0%/37.9% (*p* < 0.01) and the non-responder rates were 3.1%/27.6% (*p* < 0.01), respectively.

**Conclusions:**

The score is easily calculated from common laboratory results. It appears useful for identifying a better treatment at the time of selecting either an IL-6 or a TNF inhibitor.

## Background

Rheumatoid arthritis (RA) is a chronic inflammatory disease, characteristic of persistent synovitis and destruction of bone and cartilage in multiple joints [1]. Although its etiological causes are still unclear, the efficacy of specific proinflammatory cytokine targets clearly identifies these cytokines as relevant in RA pathogenesis. Since the first report of the tumor necrosis factor-α inhibitor (TNF-i) infliximab as the first biological disease-modifying antirheumatic drug (bDMARD) for treatment of RA in 1993 [2], bDMARDs, including several TNF-i [2–5], tocilizumab (TCZ) as an interleukin 6 (IL-6) inhibitor [6, 7], anakinra as an IL-1 inhibitor [8], abatacept (ABT) as a T cell co-stimulator inhibitor [9], and rituximab as a B cell depleting monoclonal antibody [10], have become available, and innovative progress in RA treatment has been made due to their potent clinical efficacy.

Currently, although several categories of bDMARDs are available, the preferable bDMARD for each RA case is not obvious. Therefore, TNF-i, TCZ, and ABT are basically treated as equivalent treatments in the recent disease management recommendations [11, 12]. However, it is occasionally observed that some patients who do not respond to the first biological treatment have a more obvious response to other bDMARDs. To date, many studies that focused on switching bDMARDs have been reported [5, 9, 13–16], and some authors recently suggested that switching to a bDMARD with a different mode of action may be more efficacious than switching to one that targets the same molecule [15, 16]. These studies of bDMARD switching and the aforementioned occasional cases suggest that the dominant inflammatory cytokine that should be targeted may be different in each RA patient.

For the purpose of achieving early remission and efficient medical economics, it is desirable to predict the dominant cytokine that should be inhibited before starting anticytokine therapy. The objective of this study was to develop a scoring system based on common laboratory indices that could discriminate between individuals more likely to respond to either TCZ or a TNF-i.

## Methods

### Patients

First, 45 newly diagnosed Japanese RA patients were included to estimate the relative expression of IL-6 and TNF-α mRNA in the peripheral blood before therapeutic intervention. Then, another 98 Japanese RA patients receiving TCZ or a TNF-i from 2005 to 2010 at Iizuka Hospital were studied to develop a scoring system that could discriminate between individuals more likely to respond to either TCZ or a TNF-i. We also studied an additional 228 Japanese RA patients for the validation study. They were treated with TCZ or a TNF-i at Kyushu University Hospital, Nagasaki University Hospital, Sasebo-chuo Hospital, Japanese Red Cross Okayama Hospital, and Iizuka Hospital from 2011 to 2014, and the number of patients in each setting was 31, 18, 73, 17, and 89, respectively. All of the patients fulfilled the 1987 classification criteria of the American College of Rheumatology for RA. This study was approved by the ethics committee of Kyushu University Hospital, Nagasaki University Hospital, Sasebo-chuo Hospital, Japanese Red Cross Okayama Hospital, and Iizuka Hospital, and the principles of the Helsinki Declaration were followed throughout the study. The gene expression study in 45 newly diagnosed RA patients was also approved by the ethics committee of Iizuka Hospital and was performed in accordance with the “Ethical Guidelines for Human Genome/Gene Research” published by the Japanese Ministry of Education, Culture, Sports, Science and Technology (MEXT). Informed consent was obtained from all participants.

### Analysis of gene expression level

In order to clarify the signature of gene expression at RA pathogenesis, peripheral whole blood was drawn from the 45 patients who were newly diagnosed and exposed neither to steroids nor to anti-rheumatic drugs. The samples were prepared and subjected to RNA extraction using the PAXgene system (QIAGEN, Germantown, MD, USA). Messenger RNA levels were then measured using Agilent whole human genome 60 K (Agilent Technologies, Santa Clara, CA, USA) and the log-transformed raw intensity data were normalized with a quantile algorithm. In order to evaluate the expression balance between IL-6 and TNF-α, correlation was tested between the relative mRNA levels of each gene.

### Development of a scoring system that predicts the efficacy of TCZ

Twenty-seven RA patients who were administered TCZ and 71 RA patients who were administered a TNF-i (etanercept (ETN): 36 patients, infliximab (IFX): 25 patients, adalimumab (ADA): 10 patients) at Iizuka Hospital from 2005 to 2010 were retrospectively analyzed to investigate the correlation between the efficacy of treatment and the laboratory parameters immediately before commencing treatment. Patients administered conventional DMARDs or patients with previous use of other bDMARDs were included in this analysis. In each group (TCZ group or TNF-i group), disease activity score in 28 joints using erythrocyte sedimentation rate (DAS-ESR) at week 24 and at baseline was used for calculation of the DAS ratio (= DAS-ESR at week 24/DAS-ESR at baseline) as the measure of drug efficacy. Then, correlation was tested between the DAS ratio and each laboratory test result at baseline. In this process, patients with liver dysfunction or renal dysfunction were excluded, and patients with DAS remission at baseline were also excluded. Male hemoglobin (Hb) values were multiplied by 0.88 to correct for the difference between male and female Hb levels, based on the average ratio of male and female Hb levels in similarly aged Japanese people [17]. Several items that were significantly correlated with the DAS ratio in the TCZ group but not in the TNF-i group were selected, and the cut-off values for each item were defined using receiver operating characteristic (ROC) analysis to separate the good responders to TCZ from the moderate responders and non-responders to TCZ. Then, a scoring system to predict the efficacy of TCZ was developed using these cut-off values. The validity of this scoring system was tested by applying this system to the clinical data on the aforementioned 98 patients at Iizuka Hospital.

### The validation study of the scoring system in a second set of patients

Another test was performed to validate this scoring system by using the clinical data on 228 patients (TCZ: 129 patients, TNF-i: 99 patients (ETN: 31 patients , IFX: 25 patients, ADA: 26 patients, golimumab (GLM): 13 patients, certolizumab pegol (CZP): 4 patients)). These patients were treated with TCZ or a TNF-i at Kyushu University Hospital, Nagasaki University Hospital, Sasebo-chuo Hospital, Japanese Red Cross Okayama Hospital, and Iizuka Hospital from 2011 to 2014, and the number of patients in each setting was 31, 18, 73, 17, and 89, respectively. Their laboratory tests at baseline were scored by the aforementioned scoring system, and the association between the score and the efficacy of TCZ or a TNF-i was examined.

### Statistics

The differences between two groups were analyzed using Student’s *t* test. If there were a significant difference between the variances of the two samples, the Wilcoxon rank-sum test was applied. Correlation between the *IL-6* and *TNF-α* mRNA expression levels and the DAS ratio and other continuous variables was tested using Spearman’s rank correlation. The differences between the good responder and non-responder rates in the TCZ or TNF-i groups were examined using the chi-squared (χ^2^) test. All analyses were performed by JMP statistical software (SAS Institute). *P* < 0.05 was considered statistically significant.

## Results

### Inverse correlation between *IL-6* and *TNF-α* mRNA expression in RA patients

**Fig. 1:**
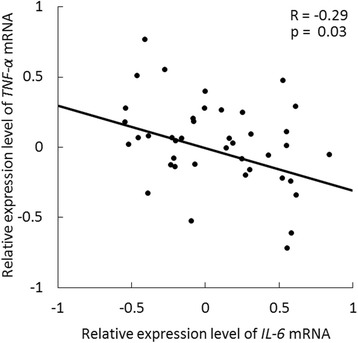
Expression levels of *IL-6* and *TNF-α* mRNA in 45 newly diagnosed rheumatoid arthritis (RA) patients. There was significant inverse correlation between *IL-6* and *TNF-α* mRNA expression levels in whole blood from 45 newly diagnosed RA patients (*R* = −0.29, *p* = 0.03)

To study the correlation between *IL-6* and *TNF-α* in RA, we measured the mRNA expression of these cytokines in peripheral blood from 45 newly diagnosed RA patients. This analysis revealed significant inverse correlation between the mRNA expression levels of *IL-6* and *TNF-α* (*R* = −0.29, *p* = 0.03), suggesting that the dominant inflammatory cytokine that should be targeted may be different in each RA patient (Fig. 1).

### Development of a scoring system that predicts the efficacy of TCZ

To aid in selecting either TCZ or a TNF-i for the treatment of RA, we aimed to develop a scoring system that reflects the dominant cytokine and predicts the efficacy of medications by analyzing common clinical data.

In this analysis, data on 98 patients treated with TCZ or a TNF-i at Iizuka Hospital from 2005 to 2010 were retrospectively analyzed. There were no significant differences between the backgrounds of patients in the IL-6 inhibitor group and the TNF-i group (Table 1). Among the 27 patients in the TCZ group, 2 patients with liver dysfunction and 2 with renal dysfunction were excluded; thus, 23 patients were included in this analysis. Among the 71 patients in the TNF-i group, in addition to 3 patients with liver dysfunction and 1 with renal dysfunction, 4 patients with DAS remission at baseline were excluded; thus, 63 patients were included in this analysis.

**Table 1:** Background data on the original 98 patients from Iizuka hospital and on an additional 228 patients from five other hospitals

	Patients from Iizuka Hospital, whose data were used to develop the scoring system (n = 98)				Patients from five other hospitals, whose data were used for the validation study (n = 228)			
	TCZ group	TNF-i group		*p* value	TCZ group	TNF-i group		*p* value
Number of patients	27	71			129	99		
		IFX	25			IFX	25	
		ADA	10			ADA	26	
		ETN	36			ETN	31	
						GLM	13	
						CZP	4	
Female, *n* (%)	24 (88.9%)	59 (83.1%)		0.74	111 (86.1%)	75 (75.8%)		0.05
Age, years (SD)	60.3 (13.4)	60.7 (14.1)		0.76	58.5 (14.0)	63.0 (13.3)		0.02
TJC (SD)	10.5 (8.5)	7.9 (7.1)		0.20	10.4 (8.3)	6.9 (6.3)		0.0019
SJC (SD)	6.4 (5.3)	5.2 (4.1)		0.36	5.3 (4.8)	6.3 (4.8)		0.06
Patient VAS, mm (SD)	53.6 (29.6)	57.3 (28.9)		0.98	47.7 (26.9)	51.3 (27.6)		0.32
ESR, mm/h (SD)	47.4 (28.6)	41.6 (28.8)		0.18	53.4 (37.5)	40.7 (28.5)		0.006
CRP, mg/dL (SD)	2.9 (3.0)	2.5 (2.4)		0.34	2.5 (3.2)	3.3 (2.8)		0.75
DAS28-ESR (SD)	5.7 (2.6)	5.2 (2.5)		0.08	5.4 (1.5)	5.0 (1.3)		0.06

Data on the original 98 patients from Iizuka hospital (2005 to 2010) were used for developing a scoring system and data on the 228 patients from Iizuka hospital (2011 to 2015) and other four hospitals were used for the validation study. Data presented are number of patients or mean unless otherwise noted. *TJC* tender joint count, *SJC* swollen joint count, *VAS* visual analog scale, *ESR* erythrocyte sedimentation rate, *CRP* C-reactive protein, *DAS28-ESR* disease activity score in 28 joints calculated by using erythrocyte sedimentation rate, *TCZ* tocilizumab, *TNF-i* tumor necrosis factor inhibitor, *IFX* infliximab, *ADA* adalimumab, *ETN* etanercept, *GLM* golimumab, *CZP* certolizumab pegol

On testing of correlation between the DAS ratio and laboratory data at baseline, there was significant correlation the DAS ratio and platelet count (Plt), Hb, aspartate aminotransferase (AST), and alanine aminotransferase (ALT), specifically in the TCZ group (Table 2). As a result of the analysis, Plt, Hb, AST, and ALT were selected, and the cut-off values were defined by ROC analysis as 381 × 103/mm3, 11.7 g/dL (male 13.2 g/dL), 16 IU/L, and 15 IU/L, respectively. Then, a scoring system was developed using these four items, and the cut-off values (1 point per item, maximum 4 points) are shown in Table 3.

**Table 2:** Correlation between improvement in the DAS and each laboratory test before treatment

Laboratory test	TCZ group		TNF-i group	
	*R*	*p* value	*R*	*p* value
Hemoglobin	0.56	0.003	−0.16	0.107
Platelet count	−0.42	0.023	0.19	0.063
Aspartate aminotransferase	0.46	0.014	0.21	0.053
Alanine aminotransferase	0.50	0.007	0.20	0.057
Lactate dehydrogenase	−0.11	0.324	0.22	0.185
Alkaline phosphatase	−0.25	0.154	−0.13	0.299
γ − Glutamiltranspeptidase	0.00	0.498	0.17	0.247
Blood urea nitrogen	0.19	0.189	−0.08	0.339
Creatinine	0.00	0.496	0.09	0.257
Total protein	−0.10	0.333	−0.28	0.078
Albumin	0.13	0.273	−0.29	0.011
Cholesterol	0.16	0.243	0.25	0.103
Low density lipoprotein	0.10	0.361	0.20	0.237
Iron	0.66	0.037	0.52	0.035
Ferritin	−0.31	0.152	0.41	0.063
Matrix metalloprotease 3	−0.13	0.283	0.16	0.151
Serum albumin A	−0.32	0.079	0.13	0.246
Rheumatoid factor	−0.06	0.403	0.18	0.310
Anticyclic citrullinated peptide antibody	0.04	0.444	0.80	0.101
Erythrocyte sedimentation rate	0.22	0.152	0.00	0.488
C reactive protein	−0.26	0.112	0.03	0.397

Correlation was tested between improvement in the DAS and each laboratory test before treatment in the tocilizumab (TCZ) group and the TNF inhibitor (TNF-i) group. There was significant correlation between the DAS ratio and hemoglobin, platelet count, aspartate aminotransferase, alanine aminotransferase, and iron in the TCZ group. The results were not similar in the TNF-i group, with the exception of correlation between the DAS ratio and iron

**Table 3:** The score table

Laboratory test			Score
Platelet count	≥381 × 10^3^/mm^3^		1
Hemoglobin (female)	≤11.7 g/dL	}	1
Hemoglobin (male)	≤13.2 g/dL		
Aspartate aminotransferase	≤16 IU/L		1
Alanine aminotransferase	≤15 IU/L		1

Each item gives 1 point. The maximum number of points is 4 if the criteria in the table for the different tests are all fulfilled

**Fig. 2:**
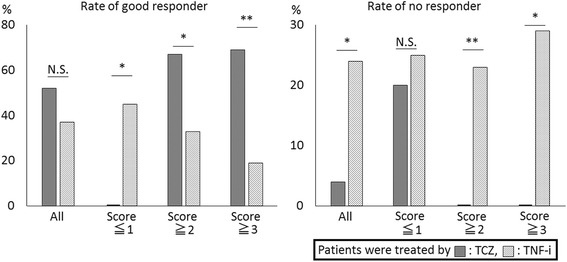
Verification test of the scoring system in 86 patients from Iizuka Hospital. Patients with 2 or more points had a significantly increased rate of good response and a lower rate of non-response to tocilizumab (*TCZ*) treatment compared with TNF inhibitor (*TNF-i*) treatment. This tendency became remarkable in patients with 3 or more points. In contrast, patients with 1 or fewer points had a significantly increased rate of good response to TNF-i treatment compared with TCZ treatment. *N.S.* not significant

Then, the 23 cases in the TCZ group and 63 cases in the TNF-i group used for the aforementioned analysis were scored using this scoring system, and the distributions of scores in each drug and each response group were compared using the European League Against Rheumatism (EULAR) response criteria. The comparison revealed that all good responders to TCZ scored 2 or more points, whereas the non-responders to TCZ and 82% of the good responders to TNF-i scored 2 or fewer points. In contrast, if the patients scored 2 or more points, the rates of being a good responder to TCZ and TNF-i were, respectively, 66.7% and 32.5% (*p* = 0.01) (the prior rates in the all of each group were 52.2% and 36.5%, respectively; *p* = 0.19) (Fig. 2). In the same group, the rates of being a non-responder to TCZ or TNF-i were, respectively, 0% and 23.3% (*p* = 0.005) (the prior rates in the all of each group were 4.3% and 23.8%, respectively; *p* = 0.02). These differences became more remarkable in patients with 3 or more points; for these patients, the rates of being a good responder to TCZ or TNF-i were, respectively, 69.2% and 19.0% (*p* = 0.003), and the rates of being a non-responder were, respectively, 0.0% and 28.6% (*p* = 0.01). In contrast, if the score was 1 or less, the rates of being a good responder to TCZ or TNF-i were 0.0% and 45.0% (*p* = 0.02). When good and moderate response to treatment was defined “condition positive” and a score of 2 or more in the TCZ group or a score of 1 or less in the TNF-i group was defined “test positive”, the predictive values were as follows: positive predictive value (PPV): 100.0%, negative predictive value (NPV): 20.0% in TCZ group, and PPV: 71.4%, NPV: 21.4% in the TNF-i group. Thus, this score seemed to supply helpful information on choosing a better treatment. There was also significant correlation between the score and the DAS ratio in the TCZ group (R = −0.60, *p* = 0.001), whereas there was no significant correlation in the TNF-i group (*R* = 0.14, *p* = 0.14).

### Usefulness of the predictive score in the second set of patients

The above verification test was thought to be insufficient because it was based on a small sample size, and the patients used in the test were the same as those used to develop the scoring system. To overcome these problems, we analyzed a second set of patients.

Therefore, a validation study was performed using the clinical data of an additional 228 patients from five hospitals. Although there were differences in the tender joint count and ESR between the TCZ and TNF-i groups, there were no significant differences between the TCZ and TNF-i groups in other clinical variables, including the DAS-ESR (Table 1). The prior rates of good response in the TCZ and TNF-i groups were 57.4% and 49.5% (*p* = 0.24), respectively, and the rates of non-response were 12.4% and 20.2% (*p* = 0.11), respectively; these data were comparable to previously reported data [18, 19]. Then, the predictive scores were calculated using the patients’ laboratory tests at baseline, and the correlation between the score and the actual efficacy of TCZ or the TNF-i was examined. To verify the universality of the scoring system, all patients were included in this analysis even though some patients had liver or renal dysfunction.

**Fig. 3:**
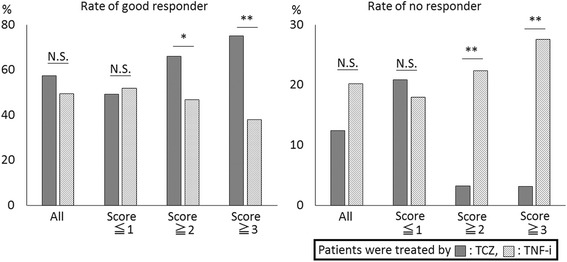
Validation of the scoring system in the second set of 228 patients from five hospitals. There were no significant differences between the prior rates of good response or non-response in the tocilizumab (*TCZ*) and TNF inhibitor (*TNF-i*) groups. Patients with a score of 2 or more points (48.1% of the TCZ group and 49.5% of the TNF-i group) had a significantly higher rate of good response and a lower rate of non-response to TCZ treatment compared with TNF-i treatment. This tendency became remarkable in patients with 3 or more points (24.8% of the TCZ group and 29.3% of the TNF-i group). In contrast, if the score was 1 or less (51.9% of the TCZ group and 50.5% of the TNF-i group), a TNF-i may be preferable because a higher rate of good response and a lower rate of non-response were observed in the TNF-i group compared with the TCZ group, although these differences were not significant: *p < 0.05, **p < 0.01. *N.S.* not significant

There was significant correlation between the score and the DAS ratio in the TCZ group (*R* = −0.27, *p* = 0.001), whereas no significant correlation was observed in the TNF-i group (*R* = 0.04, *p* = 0.36). If the score was 2 or more (48.1% of the TCZ group and 49.5% of the TNF-i group), the rates of good response in the TCZ or TNF-i group were 66.1% and 46.9% (*p* = 0.04), respectively, and the rates of non-response were 3.2% and 22.4% (*p* = 0.001), respectively; these results were especially true if the score was 3 or more (24.8% of the TCZ group and 29.3% of the TNF-i group), in which case the rates of good response in the TCZ and TNF-i groups were 75.0% and 37.9% (*p* = 0.003), respectively, and the rates of non-response were 3.1% and 27.6% (*p* = 0.004), respectively (Fig. 3). In contrast, if the score was 1 or less (51.9% of the TCZ group and 50.5% of the TNF-i group), a TNF-i may be preferable because the rates of good response in the TCZ and TNF-i groups were 49.3% and 52.0% (*p* = 0.77), respectively, and the rates of non-response were 20.9% and 18.0% (*p* = 0.70), respectively; however, these were not significant differences. Using the same definition described in the original 98 patients, predictive values of the scoring system in the additional 228 cases were as follows: PPV: 96.8% and NPV: 20.9% in the TCZ group, and PPV: 72.4% and NPV: 17.1% in the TNF-i group. The above validation study, which was applied to 228 patients from five hospitals, confirmed that the scoring system could be useful to select treatment with either TCZ or a TNF-i.

## Discussion

Currently, several types of bDMARDs are available; however, there are no guidelines on the appropriate treatment for individual RA patients according to the treatment mode of action, although there are some suggestions with respect to drug safety [20]. Therefore, TNF-inhibitors, TCZ, and ABT are treated in the same way in recent disease management recommendations [11, 12]. On the other hand, many studies focusing on switching bDMARDs showed that switching to a bDMARD with a different mode of action may be more efficacious than switching to one targeting the same molecule [15, 16], suggesting that the dominant inflammatory cytokine that should be targeted may be different in each RA patient.

However, several studies have failed to identify the dominant cytokine in a specific individual with RA [21]. In this study, we found that the relative mRNA levels of *IL-6* and *TNF-α* were inversely correlated in blood samples from 45 newly diagnosed RA patients. As the half-life of mRNA is known to be five times shorter than for proteins [22], it is possible that evaluation of the relative expression level of mRNAs may reveal timely power-balance between *IL-6* and *TNF-α* in RA. It may not be universal in each clinical situation, because the blood samples were drawn from newly diagnosed patients before any therapeutic intervention. However, it is still possible that it might be one of the explanations for the better efficacy of TCZ after inadequate response to TNF-i in some patients. The idea led us to the following study to find key laboratory indices that may reflect the power-balance between *IL-6* and *TNF-α* in RA.

Then, we found the values of Plt, iron, Hb, AST, and ALT were significantly correlated with the efficacy of TCZ treatment, and these correlations may reflect the predominant cytokine in RA pathogenesis, as described subsequently. For example, inflammatory thrombocytosis is a well-known phenomenon, and it is dependent on hepatic thrombopoietin (TPO) production stimulated by IL-6 [23, 24]. The TPO produced mediates proliferative and anti-apoptotic signals via the janus kinase (JAK) and signal transducer and activator of the transcription (STAT) pathway by binding to the cellular homolog of the myeloproliferative leukemia virus oncogene (c-MPL, CD110) receptors on the surface of megakaryocytes, leading to megakaryocyte proliferation and thrombocytopoiesis [25, 26]. Moreover, it was reported that administration of IL-6 to humans is associated with an increase in circulating platelet counts [27, 28].

IL-6 is also known to induce inflammatory anemia by the production of hepcidin. Hepcidin is a peptide hormone synthesized mainly by hepatocytes and secreted in the plasma [29]. It acts as a negative iron regulator that inhibits iron absorption from the duodenum and iron release from macrophages, leading to multiple iron metabolism disorders, including iron deficiency anemia [30, 31]. IL-6 activates the JAK/STAT signaling pathway, which activates the hepcidin promoter [31, 32]. In contrast, hepcidin production is not stimulated by TNF-α [33].

It is also a known fact that IL-6 has a hepatoprotective effect. For example, IL-6 promotes hepatic survival in a variety of liver injury models, including Fas-mediated injury and toxic damage [34–36]. It has also been shown that IL-6 is essential for liver regeneration after partial hepatectomy [37, 38]. This hepatoprotective effect of IL-6 is mediated by gp130 and JAKs, leading to the activation of STATs, which mediates anti-apoptotic and anti-necrotic signals to hepatocytes [39–42]. In addition, IL-6 is associated with liver regeneration via a mitogen-activated protein kinase (MAPK) pathway, which promotes hepatocyte proliferation [42, 43]. Moreover, it was also reported that liver regeneration was mediated by platelets [44], the production of which is promoted by IL-6 stimuli as described. Thus, IL-6 promotes hepatoprotection by various pathways. In contrast, TNF-α is a well-known, predominant mediator of hepatocyte apoptosis and liver injury by promoting caspase-8 activation and the mitochondrial death pathway via c-Jun N-terminal kinase 2 (JNK2) [45–47]. Actually in the drug information sheets, mild increased serum AST and ALT was common after TCZ treatment (≤22% for AST, ≤36% for ALT), while it was uncommon after TNF-i treatment (generally ≤5%).

While Plt, Hb, AST, and ALT were correlated with TCZ efficacy, it was an interesting finding that C-reactive protein (CRP) was not correlated with the efficacy of TCZ, and this tendency was similar in the additional 228 patients (data not shown). Recently, it was reported that baseline serum concentration of CRP might not be predictive of clinical outcomes after TCZ treatment [21]. Although the reasons for this phenomenon are unclear, such acute inflammatory markers might not be suitable for prediction because of their dispersion.

In this study, higher Plt and lower Hb, AST, and ALT levels were associated with better TCZ efficacy, and these results may be due to the aforementioned physiological functions of IL-6. Because these mechanisms are unique to IL-6, each value of Plt, Hb, AST, and ALT may reflect the cytokine balance of IL-6 and TNF-α. Recently, the efficacy of alternative IL-6 inhibitors other than TCZ has been reported [48–50]. Our scoring system was developed with the result of the efficacy of TCZ; however, the aforementioned four items depend on the physiological function of IL-6 in general, so the efficacy of other IL-6 inhibitors may be predictable using our scoring system.

A verification test of the scoring system based on the original 98 patients and an additional 228 patients revealed that patients scoring 2 or more points had a significantly higher rate of good response and a lower rate of no response to TCZ compared with a TNF-i, and this tendency became more obvious if the score was 3 or more. These results suggest TCZ could have greater efficacy in patients with a score of 2 or more (approximately 50% of all patients), and especially those with a score of 3 or more (approximately 25% of all patients), compared to those with a score less than 2. These “TCZ-sensitive” patients could be distinguished in a very simple way using our scoring system, which is easily calculated using common laboratory findings.

This study has two limitations. First, it had a relatively small sample size. It would be better to have a large sample size to develop this type of scoring system. To overcome this disadvantage, we analyzed a second set of patients. Second, this was a retrospective study, and it was difficult to secure an adequate “wash-out” period when the treatment was switched from other bDMARDs. If the score was applied after an adequate wash-out period, it is possible that the predictive value of the scoring system might be more accurate. The execution of a prospective study is desirable in the future.

## Conclusion

The gene expression analysis of newly diagnosed RA patients showed there were groups of patients with over-expression of either *IL-6* or *TNF-α*. In this study, a scoring system was developed to predict the efficacy of IL-6 inhibitor based on common laboratory tests that may reflect the dominant inflammatory cytokine. The validation study suggested that this scoring system was helpful for identifying a better treatment at the time of the selection of either an IL-6 or a TNF inhibitor.

## Abbreviations

ABT: Abatacept
ADA: Adaliumab
ALT: Alanine aminotransferase
AST: Aspartate aminotransferase
bDMARD: Biological disease-modifying antirheumatic drug
c-MPL: Cellular homolog of the myeloproliferative leukemia virus oncogene
CRP: C-reactive protein
CZP: Certolizumab pegol
DAS: Disease activity score
ESR: Erythrocyte sedimentation rate
ETN: Etanercept
EULAR: European League Against Rheumatism
GLM: Golimumab
Hb: Hemoglobin
IFX: Infliximab
IL: Interleukin
JAK: Janus kinase
JNK: c-Jun N-terminal kinase
MAPK: Mitogen-activated protein kinase
NPV: Negative predictive value
Plt: Platelet count
PPV: Positive predictive value
RA: Rheumatoid arthritis
ROC: Receiver operating characteristic
STAT: Signal transducer and activator of the transcription
TCZ: Tocilizumab
TNF: Tumor necrosis factor
TPO: Thrombopoietin
